# Novel Missense Mutations in *BEST1* Are Associated with Bestrophinopathies in Lebanese Patients

**DOI:** 10.3390/genes10020151

**Published:** 2019-02-18

**Authors:** Lama Jaffal, Wissam H. Joumaa, Alexandre Assi, Charles Helou, Christel Condroyer, Maya El Dor, Georges Cherfan, Christina Zeitz, Isabelle Audo, Kazem Zibara, Said El Shamieh

**Affiliations:** 1 Department of Biological and Environmental Sciences, Faculty of Science, Beirut Arab University, Debbieh 1107 2809, Lebanon; lama.jaffal66@gmail.com; 2 Rammal Hassan Rammal Research Laboratory, PhyToxE research group, Department of Life and Earth Sciences, Faculty of Sciences, Lebanese University, Nabatieh 1700, Lebanon; wjoumaa@ul.edu.lb; 3 Retinal Service, Beirut Eye & ENT Specialist Hospital, Beirut 1106, Lebanon; alexassi@hotmail.com (A.A.); charleshelou@hotmail.com (C.H.); georgecherfan@gmail.com (G.C.); 4 Sorbonne Université, INSERM, CNRS, Institut de la Vision, F-75012 Paris, France; christel.condroyer@inserm.fr (C.C.); christina.zeitz@inserm.fr (C.Z.); isabelle.audo@inserm.fr (I.A.); 5 Biology Department, Faculty of Sciences-I, Lebanese University, Beirut, Lebanon; mayaeldor@outlook.com; 6 CHNO des Quinze-Vingts, DHU Sight Restore, INSERM-DGOS CIC1423, F-75012 Paris, France; 7 University College London Institute of Ophthalmology, London EC1V 9EL, UK; 8 ER045, PRASE, DSST, Lebanese University, Beirut, Lebanon; 9 Department of Medical Laboratory Technology, Faculty of Health Sciences, Beirut Arab University, Beirut 1107 2809, Lebanon

**Keywords:** autosomal recessive bestrophinopathy, single vitelliform lesion, *BEST1*, mutations, Sanger sequencing

## Abstract

To identify *Bestrophin 1* (*BEST1*) causative mutations in six Lebanese patients from three families, of whom four had a presumed clinical diagnosis of autosomal recessive bestrophinopathy (ARB) and two showed a phenotype with a single vitelliform lesion, patients were subjected to standard ophthalmic examinations. In addition, *BEST1* exons and their flanking regions were amplified and sequenced by Sanger sequencing. Co-segregation and detailed bio-informatic analyses were performed. Clinical examination results were consistent with ARB diagnosis for all index patients showing multifocal vitelliform lesions and a markedly reduced light peak in the electrooculogram, including the two patients with a single vitelliform lesion. In all cases, most likely disease-causing *BEST1* mutations co-segregated with the phenotype. The ARB cases showed homozygous missense variants (M1, c.209A>G, p.(Asp70Gly) in exon 3, M2, c.1403C>T; p.(Pro468Leu) in exon 10 and M3, c.830C>T, p.(Thr277Met) in exon 7), while the two patients with a single vitelliform lesion were compound heterozygous for M1 and M2. To our knowledge, this is the first study describing mutations in Lebanese patients with bestrophinopathy, where novel biallelic *BEST1* mutations associated with two phenotypes were identified. Homozygous mutations were associated with multifocal lesions, subretinal fluid, and intraretinal cysts, whereas compound heterozygous ones were responsible for a single macular vitelliform lesion.

## 1. Introduction

Human *Bestrophin 1* (*BEST1*) (OMIM 607854), previously known as *VMD2*, is localized on chromosome 11q12.3 and consists of 11 exons [1,2]. It encodes a 585-amino acid multispan transmembrane protein that is expressed in various tissues, but predominantly localizes at the basolateral membrane of the retinal pigment epithelium (RPE) [1,2]. BEST1 is associated with calcium-activated chloride ion channel activity in epithelial cells [3]. Chloride currents generated by this channel are found to be volume-sensitive, suggesting that they are involved in the regulation of the volume of RPE cells [4,5]. It was also shown that these chloride channels are highly permeable to bicarbonate ion (HCO_3_^−^) and may play a role in the conductance of HCO_3_^−^ in RPE cells [6].

Mutations in *BEST1* have been associated with a range of clinically recognized ocular disorders in humans, collectively termed as bestrophinopathies [7]. This group includes the autosomal dominant forms as the classic and well known Best vitelliform macular dystrophy (BVMD or Best disease; OMIM 153700) [1,2], adult-onset vitelliform macular dystrophy (AVMD; OMIM 153700), vitreoretinochoroidopathy (ADVIRC; OMIM 193220) [8], and autosomal recessive bestrophinopathy (ARB; OMIM 611809) [9]. Along with bestrophinopathies, mutated *BEST1* was reported in patients with retinitis pigmentosa-50 (RP50; OMIM 613194) [10]. However, Leroy (2012) commented that although the phenotypes of the patients reported by Davidson et al. (2009) were all labeled “retinitis pigmentosa” initially, the illustrations of the retinal phenotypes in the paper were highly suggestive of either autosomal dominant vitreoretinochoroidopathy (193220) or ARB (611809) [11]. The hallmark of these BEST1-related dystrophies is a severely reduced electro-oculorgam (EOG) light rise with no or minimal to mild full-field electroretinogram (ERG) abnormalities in keeping with primary RPE dysfunction. Patients with ARB show a severe reduction in the EOG light rise similar to that seen in both BVMD and ADVIRC [9]. Uniquely, they show multifocal vitelliform lesions, with subretinal fluid and intraretinal cysts, scattered over the posterior pole of the retina [9]. In addition, their full-field ERG responses are usually decreased and delayed for both the cone and rod systems [9].

Although *BEST1* mutations were initially associated with autosomal dominant inheritance [1,2], Schatz et al. reported an autosomal recessive mode of inheritance in two Swedish patients in 2006, but the phenotype was defined as “a variant form of Best macular dystrophy” [12]. Two years later, Burgess et al. designated this phenotype as a “distinct retinopathy” and used the term “autosomal recessive bestrophinopathy” (ARB) for the first time [9]. Subsequently, ARBs were reported in many other cases [13,14,15]. Nevertheless, those findings do not negate the fact that autosomal dominant segregation of *BEST1* mutations remains the most common form; while, autosomal recessive mode is much rarer with more than 24 published papers (Supplementary Table S1) [9,10], and an extremely low prevalence of <1:1,000,000 [16]. The age of onset of autosomal recessive cases is usually earlier than for dominant cases [17,18]. The presence of biallelic (homozygous or compound heterozygous) *BEST1* mutations usually abolishes chloride conductance [9].

A large number of *BEST1* mutations associated with bestrophinopathies have been reported in previous studies conducted on various populations from different ethnic groups, but none on near Eastern populations. In the present study, we aimed to identify causative *BEST1* mutations in six Lebanese patients from three families with a presumed diagnosis of ARB and showing two phenotypes; one with multifocal lesions, the other with a single macular vitelliform lesion. 

## 2. Materials and Methods

### 2.1. Patient Recruitment and Ethics Statement

Affected individuals were recruited at Beirut Eye and ENT Specialist Hospital (Beirut, Lebanon, after being clinically, but not genetically, diagnosed with bestrophinopathies. Written informed consent was obtained from each index patient according to the tenets of the Declaration of Helsinki. In addition, the institutional review board of Beirut Arab University approved the study, under the IRB code: 2017H-0030-HS-R-0208.

#### Clinical Examinations

All recruited patients underwent a clinical ophthalmic examination, including a detailed family history, fundus photography, fundus autofluorescence, fundus fluorescein angiography (FFA), and optical coherence tomography (OCT), in addition to electrophysiological tests involving ERG and EOG. Fundus autofluorescence imaging and fluorescein angiography were performed using the TRC-50DX machine (Topcon, Tokyo, Japan), the OCT images were obtained using the 3D OCT-2000 (Topcon, Japan), and the electrophysiological testing was recorded with the Vision Monitor Mon2010E (Metrovision, Pérenchies, France). 

### 2.2. Molecular Analysis and Mutations Detection

DNA extraction: DNA extraction was performed on whole blood samples obtained from all participants, using a DNA extraction kit from Qiagen (QIAamp DNA Mini Kit; Hilden, Germany) according to the manufacturer’s protocol. A Qubit 3.0 fluorometer (Thermo Fisher Scientific; Shah Alam, Malaysia) was used to quantify the DNA extracts using the Qubit dsDNA BR Assay Kit (Thermo Fisher Scientific; Shah Alam, Malaysia).

Polymerase chain reaction (PCR) and Sanger sequencing: PCR was performed using the thermal cyclers (Veriti, Applied Biosystems, and T100, Biorad, Kaki Bukit, Singapore). Primers were designed to flank each of the 11 exons and the exon-intron boundaries of *BEST1*. PCR products were then purified with a QIAquick PCR Purification Kit (Qiagen, Hilden, Germany). Purified PCR products were sequenced from both forward and reverse directions. More details about the primers and the reaction conditions are available upon request. Sequences were obtained using a DNA analyzer (Applied Biosystems 3730xl DNA Analyzer, Courtaboeuf, Les Ulis, France)**.**

Sequence analysis for mutation detection: The obtained sequences were analyzed using the software, SeqScape v2.6 (for families 1 and 2), Applied Biosystems, and the Chromas Lite 2.1 software (for family 34), and compared to the reference sequence of human *BEST1* (NM: 004183.3).

Pathogenicity assessment of candidate variants: The University of California, Santa Cruz UCSC genome browser was used to obtain the degree of conservation of the candidate variant across different species [19]. The 1000 genomes database [20], Ensembl GRCh37 genome browser [21], Genome Aggregation Database (gnomAD), and Exome Aggregation Consortium database (ExAC) [22] were used to determine the minor allele frequency (MAF) of candidate variants. Scale-invariant feature transform (SIFT) [23], polymorphism phenotyping v2 (PolyPhen-2**)** [24], and Mutation Taster2 [25] were used to predict the possible impact of the detected amino acid substitutions on the BEST1 function.

Co-segregation analysis: When candidate mutations were revealed in index patients, a familial co-segregation analysis was performed in available family members.

#### Genotype-Phenotype Associations

The Human Gene Mutation Database [26], Leiden Open Variation Database [27] (http://www.lovd.nl/2.0/index_list.php?search_symbol=best1), and Online Mendelian Inheritance in Man [28] and PubMed were used to determine whether the detected variants were novel or previously described to be associated with ARB.

## 3. Results

### 3.1. Ophthalmic Data

**Figure 1:**
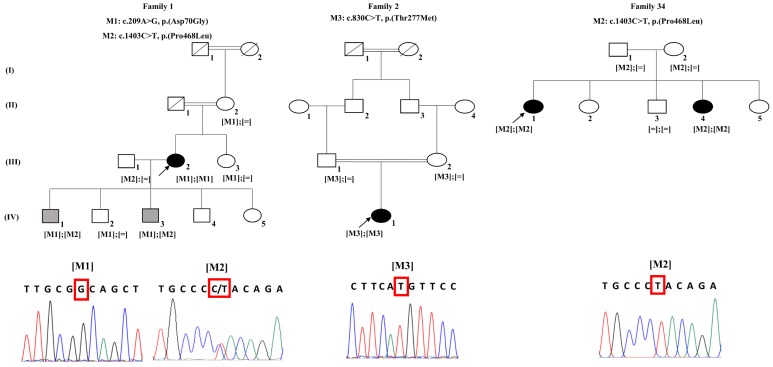
Pedigrees of three families diagnosed with autosomal recessive bestrophinopathy. Chromatograms for index patients were also shown: [M1] belongs to F1: III.2; [M2] in family 1 belongs to F1: IV.1; [M3] belongs to F2: IV:1; [M2] in family 34 belongs to F34: II.1. White symbols indicate unaffected patients. Black symbols indicate multifocal lesions, subretinal fluid, and intraretinal cysts (F1: III.2). Gray symbols indicate a phenotype with a single vitelliform lesion (F1: IV.1 and F1: IV.3). Square and round symbols represent males and females, respectively. The slash indicates deceased individuals. The black arrows indicate the probands (F1: III.2, F2: IV.1, F34: II.1). Double horizontal lines represent consanguineous marriages. M signifies mutation.

**Figure 2:**
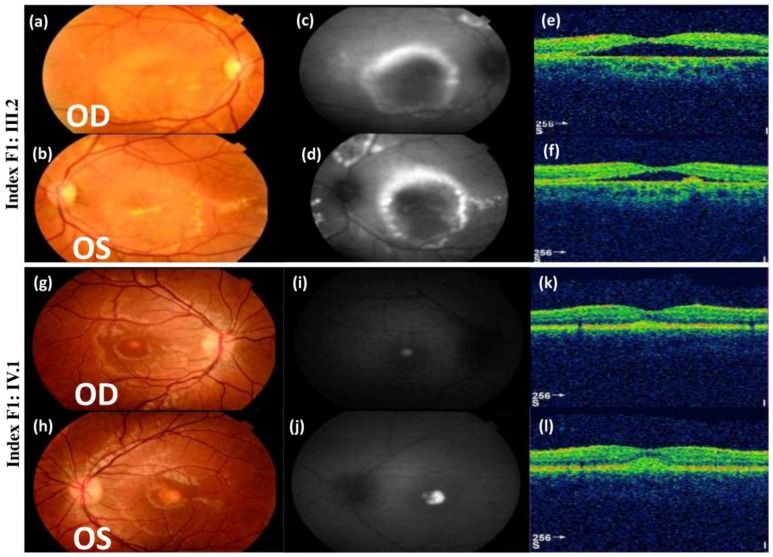
Color fundus photographs (**a**,**b**,**g**,**h**), auto-fluorescence pictures (**c**,**d**,**i**,**j**), and optical coherence tomography scans (**e**,**f**,**k**,**l**) of indexes F1: III.2 and F1: IV.1. Fundus photographs of F1: III.2 showed multifocal vitelliform lesions, subretinal fluid, and intraretinal cysts. Colour fundus photographs of F1: IV.1 showed the presence of a single vitelliform lesion in the posterior pole of both eyes that was hyperautofluorescent on autofluorescence imaging, Optical coherence tomography (OCT) scans showed hyperreflective material at the fovea. OD = oculus dexter; OS = oculus sinister.

Index F1: III.2 (a 41-year-old female with no family history) belongs to a family with two generations of consanguinity as both of her parents and maternal grand-parents were first degree cousins (Figure 1). She started experiencing reduced vision at the age of 17, but was not diagnosed with ARB until the age of 31. Fundus photographs of this patient showed multivitelliform lesions, subretinal fluid, and intraretinal cysts on the autofluorescence imaging. There was additional vitelliform lesions outside the vascular arcades. In addition, OCT showed a central serous detachment with some hyperreflective material at the macula and intra retinal cysts in the paramacular region (Figure 2). EOG showed no light rise and a reduced Arden ratio of 1.7 on the right and 1.5 on the left, which are below the normal value (>1.8); ERG results were within the normal range (data not shown).

F1: IV.1 (a 14-year-old male) started to complain of problems in vision at the age of 11. Colour fundus photographs showed the presence of a single vitelliform lesion in the posterior pole that was hyperautofluorescent on the autofluorescence imaging. Moreover, OCT showed hyperreflective material at the fovea (Figure 2). The EOG recording did not reveal any light rise with a severely reduced Arden ratio for both eyes (data not shown). Similarly, F1: IV.3 (6 year old male) showed a single vitelliform lesion at the center of the macula confirmed on OCT (Supplementary Figure S1). In contrast, the clinical assessment of the unaffected members in F1 revealed that F1: III.1, F1: III.3, F1: IV.2, F1: IV.4, and F1: IV.5 have normal fundus and OCT images (Supplementary Figure S1).

Index F2: IV.1, resulting from a consanguineous marriage (Figure 1), showed multifocal vitelliform lesions on fundus examination at the age of 6 years (Supplementary Figure S2). EOG also revealed an absent light rise with very reduced Arden ratios of 1.3 on the right and 1.2 on the left. Similar abnormalities were detected in the fundus photographs and OCT scans between index patient F34: II.1 and index F1: III.2 (Supplementary Figures S1 and S3). Clinical assessment of the unaffected mother, F2: III.2, showed normal fundus photographs and OCT (Supplementary Figure S2).

In addition, indexes F34: II.1 and F34: II.4 showed multifocal vitelliform lesions on fundus examination (Supplementary Figure S3), and very reduced Arden ratios consisting of 1.06 on the right and 1.01 on the left. Their OCT findings were also abnormal whereas the unaffected parents F34: I.1 and F34: I.2 showed normal results (Supplementary Figure S3).

### 3.2. Genetic Findings

**Family 1 (F1)**: Index F1: III.2 exhibited a homozygous mutation (M1); c.209A>G; p.(Asp70Gly), rs749295558 in exon 3 (Figure 1). Co-segregation analysis showed that her second son (F1: IV.2) and sister (F1: III.3) were both heterozygous for this mutation (Figure 1), with no symptoms (Table 1). Her elder son (F1: IV.1) also carried the same heterozygous mutation (Figure 1). Mutation M1 was shown to be rare heterozygous in ExAC and gnomAD populations (G = 0.00002 and 0.000008, respectively), affecting a highly conserved amino acid residue across species with only two exceptions according to the UCSC genome browser. It was also predicted to be probably damaging and deleterious according to PolyPhen-2 and SIFT, respectively. Most importantly, this mutation was not reported in the literature according to HGMD, LOVD, and OMIM (Table 2). Interestingly the clinical tests performed for the son, F1: IV.1, during our study revealed that he also presented a vitelliform lesion in both eyes (Figure 2). Thus, the remaining *BEST1* exons were screened and the presence of a second causative mutation (M2); c.1403C>T; p.(Pro468Leu), rs747043918 in exon 10 was identified in this individual. The mutation was shown to be extremely rare and heterozygous in ExAC and gnomAD (T = 0.000008 and 0.000004, respectively). It affected a well-conserved amino acid residue across species with no exceptions and was predicted to be deleterious and disease-causing (Table 2). M2 was not previously reported. Co-segregation analyses revealed that M1 and M2 co-segregated adequately with the phenotypes: The patient with multifocal vitelliform lesion (F1: III.2) was homozygous for M1, while patients with single vitelliform lesions (F1: IV.1 and F1: IV.3) were compound heterozygous for M1 and M2 (Figure 1). Other unaffected family members (F1: II.2, F1: III.1, F1: III.3, and F1: IV.2) were either heterozygous for M1 or M2 or wild type (Figure 1 and Supplementary Figure S4), making M2 in the heterozygous state not disease causing. 

**Family 2 (F2)**: Index F2: IV.1 presented the homozygous mutation (M3); c.830C>T; p.(Thr277Met), rs775791299 in exon 7 (Figure 1). Both unaffected consanguineous parents were heterozygous for M3 that was also shown to be rare heterozygous in ExAC and gnomAD (T = 0.0000082 in both databases). Thus, it affected a well-conserved residue across species with no exceptions. Prediction tools revealed it was deleterious and disease-causing (Table 2). Heterozygous members (father; F2: III.1/mother; F2: III.2) were asymptomatic and did not report any vision problem (Table 1). This mutation has previously been reported in association with ARB [29,30].

**Family 34 (F34):** The two affected sisters, F34: II.1 and F34: II.4, presented the same homozygous mutation (M2); c.1403C>T; p.(Pro468Leu), rs747043918 in exon 10 (Figure 1). Both unaffected parents were heterozygous carriers for this mutation while the unaffected brother was homozygous for the reference sequence.

## 4. Discussion

To date, more than 250 distinct mutations were identified in *BEST1* in various forms of bestrophinopathies [16]. In the current study, three *BEST1* missense mutations were identified, all of which are very rare and never homozygous in 1000 genomes (2504 sequences), gnomAD (123,136 exome sequences and 15,496 whole genome sequences), and ExAC browser (60,706 exome sequences). The literature review showed that the two mutations, M1 and M2; c.209A>G; p.(Asp70Gly) and c.1403C>T; p.(Pro468Leu), respectively, were novel. However, mutation M3; c.830C>T; p.(Thr277Met) has already been described to cause ARB [29,30]. In contrast to dominant mutations leading to a single vitelliform lesion, biallelic mutations causing ARB are characterized by the presence of multifocal vitelliform lesions, with subretinal fluid and intraretinal cysts, scattered all over the posterior pole of the retina [9]. In addition, full-field ERG examination may show a reduction in the cone and rod responses along with an absence or markedly reduced light peak in EOG. Interestingly, in all our patients with biallelic *BEST1* mutations, the detected biallelic mutations were found to be associated with two phenotypes: One with multifocal lesions, subretinal fluid, and intraretinal cysts in F1: III.2, F2: IV.1, F34: II.1, and F34: II.4, or a single macular vitelliform lesion in F1:IV.1 and F1:IV.3. The novelty of two mutations in only three families suggests that many more ARB mutations are yet to be discovered. This is mainly due to the high rates of consanguinity that increase the incidence of recessively inherited diseases through a few number of “common” mutations rather than a large prevalence of rare mutations [31].

In the first family, EOG results suggested that the phenotype is associated with *BEST1* mutations. Genetic screening of *BEST1* showed that index F1: III.2, with presumed diagnosis of ARB, harbored the novel causative M1 mutation p.(Asp70Gly) in a homozygous state. This was further confirmed by the co-segregation analyses: Individuals F1: II.2, F1. III.1, F1. III.3, and F1: IV.2 were all healthy heterozygous carriers without any clinical manifestations (Supplementary Figure S1), which confirms the autosomal recessive mode of inheritance for F1: III.2. Remarkably, the two sons of individual F1: III.2, F1: IV.1 and F1: IV.3, carrying the M1 mutation heterozygously, only had a single vitelliform lesion. However, heterozygous M1 does not seem to be disease causing since F1: II.2, III.3, nor IV2 showed such a phenotype. Interestingly, subsequent sequencing of the remaining exons revealed indeed a second mutation, M2; p.(Pro468Leu). This single vitelliform lesion phenotype has also been reported in several articles in patients carrying biallelic *BEST1* mutations [14,30,32]. Similar to our case, two other ARB homozygous mutations (p.R13H and p.A195V) in Chinese patients were reported to cause a single vitelliform lesion if they occurred in a compound heterozygous state with other mutations [30]. Of note, most of these previous articles reported mutations leading to an ARB in one family and a single vitelliform lesion in another one, whereas in the present report, both phenotypes co-exist in the same family. This highlights the high variability of intrafamilial clinical expressivity related to *BEST1* mutations. Schatz et al. reported two siblings both having the compound heterozygous mutations, p.(Y29ter)/p.(R141H), but one had a central single lesion while the other had more widespread changes [12]. The two compound heterozygous patients of our family, F1, showing single vitelliform lesions are still quite young, thus we cannot exclude that they develop extramacular lesions at an older age [33]. Another example that highlights the complexity of genotype-phenotype correlation with *BEST1* mutations is reflected by the most recurrent mutation; p.(R141H). This mutation is known to be associated with ARB when homozygous [17]; in contrast, when heterozygous, the mutation is reported with either a single vitelliform lesion [34] or as being asymptomatic [9]. Some patients with single vitelliform lesions may carry compound heterozygous mutations in conjunction with p.(R141H), such as the case of p.(R141H) and p.(D312N) [17]. This implies that other environmental and genetic factors might affect this phenotype [35,36].

In the second family, index F2: IV.1 presented the M3 mutation; c.830C>T; p.(Thr277Met) in a homozygous state while both of her asymptomatic consanguineous parents were heterozygous. These findings support the autosomal recessive pattern of inheritance and confirm that this biallelic mutation segregated only with ARB. This is consistent with the same recently reported mutation by Zaneveld et al. (2015), in a 33-year-old Canadian female [29]. Although the aim of the latter study was to analyze a cohort of patients with a presumed clinical diagnosis of Stargardt macular dystrophy (STGD), the detection of the M3 mutation in the *BEST1* of this patient resulted in a re-diagnosis with ARB [29]. This confirms the importance of molecular testing to determine an accurate diagnosis in complex inherited retinal dystrophy cases [37]. The same mutation was also reported later by Tian et al. (2017) in a Chinese ARB patient, as a compound heterozygous state along with p.(Leu109Tyr) mutation [30].

In F34, both affected sisters, F34: II.1 and F34: II.4, presented the novel homozygous mutation, M2; c.1403C>T; p.(Pro468Leu). Similarly to families one and two, the autosomal recessive mode of inheritance was confirmed by the co-segregation analysis since all available unaffected members were either heterozygous carriers or normal wild type.

Taken together, the data obtained from all three families suggests that M1, M2, and M3 are associated with different forms of vitelliform lesions depending on their status; multifocal vitelliform lesions were found in cases with homozygous variants and single vitelliform lesions were found in the compound heterozygous state.

Our understanding of the molecular mechanisms underlying the clinical manifestations of BEST1 associated pathologies is still limited and shows contradicting findings [15,16,36,38]. Mechanistic data from Davidson et al. [15] and Uggenti et al. [36] showed that the majority of recessive mutations leading to ARB (except p.(D312N) and p.(V317M)) are degraded by the proteasome and not the lysosome [15,36]. More recently, ARB mutations were demonstrated to accelerate the BEST1 degradation process in the endoplasmic reticulum (ER), through a mechanism known as endoplasmic-reticulum-associated protein degradation (ERAD), thereby favoring a decreased assembly of the homo-pentameric BEST1 chloride channel [16]. In contrast, heterozygous mutations causing single vitelliform lesions will escape ERAD and instead are recognized by a post-ER quality control mechanism at the Golgi complex via the endo-lysosomal degradation pathway, which helps them to exert their dominant-negative potential [16] in dominant cases. This is not the case for a BVMD mutation (R218C) that has the same expression and turnover as wildtype BEST1 protein [16]. Nothing is known yet about compound heterozygous mutations. Mutation M1 is located in the second segment of the protein, close to a narrowed region forming the neck of the ion pore of BEST1 channel [39]. Mutations associated with eye disease are particularly prevalent in or around the neck of the pore [39]. This neck was shown to function as a Ca^2+^ dependent gate that permits or prevents anion permeation in the channel [40]. M2 is located in the cytoplasmic C-terminus part of the protein and specifically affects the highly-conserved proline (P468) found at the beginning of a cluster of proline-rich motifs falling between amino acid positions 468 and 486 that is conserved among many species [41]. This C-terminus cluster identified by Milenkovic et al. may have a possible influence on the pore formation and the gating properties of Ca^2+^ channels [41]. Mutation M3 affects the amino acid, threonine 277, that was reported to exert a stabilizing interaction with one of the anion binding sites within the ion pore, which supports the deleterious effect of M3 [39].

## 5. Conclusions

In conclusion, this study identified three *BEST1* mutations in six Lebanese patients from three families. This step expanded the mutational spectrum associated with this disorder and the highly phenotypic variability associated with *BEST1* mutations. Furthermore, this study is the first of its kind focused on understanding the genetics of bestrophinopathies in near-eastern populations. This is significantly important for the accurate diagnosis of ARB relevant to genetic counseling and enrollment in future clinical trials.

## Figures and Tables

**Table 1:** Clinical results identified in three Lebanese families.

Index (Status)	Age at Onset	Fundus (Phenotype of Viteliform Lesion)	OCT	EOG Arden Ratio (O.D./O.S.)	ERG
F1: III.2 (affected)	17	Ring-like deposition of yellowish material around the macula (multifocal lesions)	Central serous detachment with some hyperreflective material	1.7/1.5	Normal
F1: IV.1 (affected)	11	Bilateral vitelliform lesions in the posterior pole of the macula (single vitelliform lesion)	Hyperreflective material at the fovea	1.3/1.3	-
F1: IV.3 (affected)	6	Bilateral vitelliform lesions in the posterior pole of the macula (single vitelliform lesion)	Hyperreflective material at the fovea	1.1/1.1	-
F1: III.1 (unaffected)	-	Normal	Normal	-	-
F1: III.3 (unaffected)	-	Normal	Normal	-	-
F1: IV.2 (unaffected)	-	Normal	Normal	-	-
F2: IV.1 (affected)	6	Multifocal vitelliform lesions	Central serous detachment with some hyperreflective material	1.3/1.2	-
F2: III.2 (unaffected)	-	Normal	Normal	-	-
F34: II.1 (affected)	15	Multifocal vitelliform lesions	Central serous detachment with some hyperreflective material	1.01/1.03	Normal
F34: II.4 (affected)	11	Multifocal vitelliform lesions	Central serous detachment with some hyperreflective material	1.06/1.01	-
F34:I.1 (unaffected)	-	Normal	Normal	-	-
F34: I.2 (unaffected)	-	Normal	Normal	-	-

**Table 2:** *BEST1* mutations identified in three Lebanese families.

Index (Status)	Age at Onset	Exon(s)	Status	rs ID and Nucleotide Exchange	Amino Acid Change	Frequencies	PolyPhen-2	SIFT	Mutation Taster
F1: III.2 (affected)	17	3	Homozygous	M1: rs749295558, c.209A>G	M1: p.(Asp70Gly)	M1: 0.00002 (ExAc) 0.00008 (GnomAD) (never homozygous); M2: 0.000008 (ExAc) 0.000004 (GnomAD) (never homozygous)	M1 and M2: Probably damaging	M1 and M2: Deleterious (score < 0.05)	M1 and M2: Disease-causing (*p* = 0.99)
F1: IV.1 (affected)	11	3 & 10	Compound Heterozygous	M1: rs749295558, c.209A>G M2: rs747043918, c.1403C>T	M1: p.(Asp70Gly) M2: p.(Pro468Leu)				
F1: IV.3 (affected)	6	3 & 10	Compound Heterozygous	M1: rs749295558, c.209A>G M2: rs747043918, c.1403C>T	M1: p.(Asp70Gly) M2: p.(Pro468Leu)				
F1: II.2 (unaffected)	-	3	Heterozygous	M1: rs749295558, c.209A>G	M1: p.(Asp70Gly)				
F1: III.1 (unaffected)	-	10	Heterozygous	M2: rs747043918, c.1403C>T					
F1: III.3 (unaffected)	-	3	Heterozygous	M1: rs749295558, c.209A>G					
F1: IV.2 (unaffected)	-	3	Heterozygous	M1: rs749295558, c.209A>G					
F2: IV.1 (affected)	6	7	Homozygous	M3: rs775791299, c.830C>T	p.(Thr277Met)	M3: 0.0000082 (ExAc) 0.0000082 (GnomAD) (never homozygous)	Benign	Deleterious (score < 0.05)	Disease-causing (*p* = 0.99)
F2: III.1 (unaffected)	-		Heterozygous	M3: rs775791299, c.830C>T					
F2: III.2 (unaffected)	-		Heterozygous	M3: rs775791299, c.830C>T					
F34: II.1 (affected)	15	10	Homozygous	M2: rs747043918, c.1403C>T	p.(Pro468Leu)	M2: 0.000008 (ExAc) 0.000004 (GnomAD) (never homozygous)	Probably damaging	Deleterious (score < 0.05)	Disease-causing (*p* = 0.99)
F34: II.4 (affected)	11		Homozygous	M2: rs747043918, c.1403C>T					
F34: I.1 (unaffected)	-		Heterozygous	M2: rs747043918, c.1403C>T					
F34: I.2 (unaffected)	-		Heterozygous	M2: rs747043918, c.1403C>T					
F34: II.3 (unaffected)	-		WT	-	-	-	-	-	-

WT: Wild-type.

## Supplementary Materials

The following are available online at https://www.mdpi.com/2073-4425/10/2/151/s1, Figure S1: Color fundus photographs (a,b), auto-fluorescence pictures (c,d), red free fundus photographs (c’,d’), optical coherence tomography scans; OCT (e,f) of family members (F1: III.1, F1: III.3, F1: IV.2, F1: IV.3, F1: IV.4, F1: IV.5). OD = oculus dexter; OS = oculus sinister, Figure S2: Color fundus photographs (a–d), optical coherence tomography scans; OCT; (e,f) of index F2: IV.1. OD = oculus dexter; OS = oculus sinister, Figure S3: Color fundus photographs (a,b), auto-fluorescence pictures; (c,d), optical coherence tomography scans; OCT; (e,f) of indexes (F34: II.1 - F34: II.4) and their parents (F34: I.2 - F34: I.1). OD = oculus dexter; OS = oculus sinister, Figure S4: Chromatograms of additional affected and unaffected family members from Families 1, 2 and 34, Table S1: List of published papers on autosomal recessive bestrophinopathy, Table S2: List of all *BEST1* variants detected in affected individuals of each family.
