# Supplementary material for: Novel Missense Mutations in BEST1 Are Associated with Bestrophinopathies in Lebanese Patients
Source: Genes (Basel). 2019 Feb 18;10(2):151. doi: 10.3390/genes10020151 (PMC6409913; doi:10.3390/genes10020151)
Supplement: Supplementary file 1 [file genes-10-00151-s001.pdf]

Supplementary

Table S1. List of published papers on autosomal recessive bestrophinopathy.

Study Title	Authors	Journal and Year of Publication
Biallelic mutation of best1 causes a distinct retinopathy in humans.	Burgess, R.; Millar, I.D.; Leroy, B.P.; Urquhart, J.E.; Fearon, I.M.; De Baere, E.; Brown, P.D.; Robson, A.G.; Wright, G.A.; Kestelyn, P., et al.	Am J Hum Genet **2008**, 82, 19-31
The spectrum of ocular phenotypes caused by mutations in the best1 gene.	Boon, C.J.; Klevering, B.J.; Leroy, B.P.; Hoyng, C.B.; Keunen, J.E.; den Hollander, A.I.	*Progress in retinal and eye research* **2009**, *28*, 187-205.
Missense mutations in a retinal pigment epithelium protein, bestrophin-1, cause retinitis pigmentosa.	Davidson, A.E.; Millar, I.D.; Urquhart, J.E.; Burgess-Mullan, R.; Shweikh, Y.; Parry, N.; O'Sullivan, J.; Maher, G.J.; McKibbin, M.; Downes, S.M., et al	Am J Hum Genet **2009**, 85, 581-592.
A synonymous codon variant in two patients with autosomal recessive bestrophinopathy alters in vitro splicing of best1.	Davidson, A.E.; Sergouniotis, P.I.; Burgess-Mullan, R.; Hart-Holden, N.; Low, S.; Foster, P.J.; Manson, F.D.; Black, G.C.; Webster, A.R.	Mol Vis **2010**, 16, 2916-2922.
Functional characterization of bestrophin-1 missense mutations associated with autosomal recessive bestrophinopathy.	Davidson, A.E.; Millar, I.D.; Burgess-Mullan, R.; Maher, G.J.; Urquhart, J.E.; Brown, P.D.; Black, G.C.; Manson, F.D.	Invest Ophthalmol Vis Sci **2011**, 52, 3730-3736.
Autosomal recessive bestrophinopathy: New observations on the retinal phenotype - clinical and molecular report of an italian family.	Guerriero, S.; Preising, M.N.; Ciccolella, N.; Causio, F.; Lorenz, B.; Fischetto, R.	Ophthalmologica. **2011**, 225, 228-235.
Ocular phenotypes associated with biallelic mutations in best1 in italian patients.	Sodi, A.; Menchini, F.; Manitto, M.P.; Passerini, I.; Murro, V.; Torricelli, F.; Menchini, U.	*Mol Vis* **2011**, *17*, 3078-3087.
Childhood-onset autosomal recessive bestrophinopathy.	Borman, A.D.; Davidson, A.E.; O'Sullivan, J.; Thompson, D.A.; Robson, A.G.; De Baere, E.; Black, G.C.; Webster, A.R.; Holder, G.E.; Leroy, B.P., et al.	Arch Ophthalmol **2011**, 129, 1088-1093.
[autosomal recessive bestrophinopathy (arb): A clinical and molecular description of two patients at childhood].	Preising, M.N.; Pasquay, C.; Friedburg, C.; Bowl, W.; Jager, M.; Andrassi-Darida, M.; Lorenz, B.	Klin Monbl Augenheilkd **2012**, 229, 1009-1017.
Phenotype and genotype of patients with autosomal recessive bestrophinopathy.	MacDonald, I.M.; Gudiseva, H.V.; Villanueva, A.; Greve, M.; Caruso, R.; Ayyagari, R.	Ophthalmic Genet **2012**, 33, 123-129.
Nonsense-mediated decay as the molecular cause for autosomal	Pomares, E.; Bures-Jelstrup, A.; Ruiz-Nogales, S.;	Invest Ophthalmol Vis Sci

recessive bestrophinopathy in two unrelated families.	Corcostegui, B.; Gonzalez-Duarte, R.; Navarro, R.	**2012**, 53, 532-537.
Autosomal recessive bestrophinopathy: Differential diagnosis and treatment options.	Boon, C.J.; van den Born, L.I.; Visser, L.; Keunen, J.E.; Bergen, A.A.; Booij, J.C.; Riemslag, F.C.; Florijn, R.J.; van Schooneveld, M.J.	Ophthalmology **2013**, 120, 809-820.
Ocular phenotype analysis of a family with biallelic mutations in the best1 gene.	Sharon, D.; Al-Hamdani, S.; Engelsberg, K.; Mizrahi-Meissonnier, L.; Obolensky, A.; Banin, E.; Sander, B.; Jensen, H.; Larsen, M.; Schatz, P.	*Am J Ophthalmol* **2014**, *157*, 697-709 e691-692.
Screening for best1 gene mutations in chinese patients with bestrophinopathy.	Tian, R.; Yang, G.; Wang, J.; Chen, Y.	*Mol Vis* **2014**, *20*, 1594-1604.
A novel best1 mutation in autosomal recessive bestrophinopathy.	Lee, C.S.; Jun, I.; Choi, S.I.; Lee, J.H.; Lee, M.G.; Lee, S.C.; Kim, E.K	Invest Ophthalmol Vis Sci **2015**, 56, 8141-8150.
New best1 mutations in autosomal recessive bestrophinopathy.	Fung, A.T.; Yzer, S.; Goldberg, N.; Wang, H.; Nissen, M.; Giovannini, A.; Merriam, J.E.; Bukanova, E.N.; Cai, C.; Yannuzzi, L.A., et al.	Retina **2015**, 35, 773-782.
Clinical and genetic findings of autosomal recessive bestrophinopathy in japanese cohort.	Nakanishi, A.; Ueno, S.; Hayashi, T.; Katagiri, S.; Kominami, T.; Ito, Y.; Gekka, T.; Masuda, Y.; Tsuneoka, H.; Shinoda, K., et al.	Am J Ophthalmol **2016**, 168, 86-94.
Detailed analysis of family with autosomal recessive bestrophinopathy associated with new best1 mutation.	Kubota, D.; Gocho, K.; Akeo, K.; Kikuchi, S.; Sugahara, M.; Matsumoto, C.S.; Shinoda, K.; Mizota, A.; Yamaki, K.; Takahashi, H., et al.	Doc Ophthalmol **2016**, 132, 233-243.
Biallelic mutations in the best1 gene: Additional families with autosomal recessive bestrophinopathy.	Wivestad Jansson, R.; Berland, S.; Bredrup, C.; Austeng, D.; Andreasson, S.; Wittstrom, E	Ophthalmic Genet **2016**, 37, 183-193.
Flat anterior chamber after trabeculectomy in secondary angle-closure glaucoma with best1 gene mutation: Case series.	Zhong, Y.; Guo, X.; Xiao, H.; Luo, J.; Zuo, C.; Huang, X.; Huang, J.; Mi, L.; Zhang, Q.; Liu, X.	PLoS One **2017**, 12, e0169395.
A unique case series of autosomal recessive bestrophinopathy exhibiting multigenerational inheritance.	Hardin, J.S.; Schaefer, G.B.; Sallam, A.B.; Williams, M.K.; Uwaydat, S.	Ophthalmic Genet **2017**, 38, 570-574.
Screening of best1 gene in a chinese cohort with best vitelliform macular dystrophy or autosomal recessive bestrophinopathy.	Tian, L.; Sun, T.; Xu, K.; Zhang, X.; Peng, X.; Li, Y.	Invest Ophthalmol Vis Sci **2017**, 58, 3366-3375.
Ten-year follow-up after bilateral submacular neovascular membrane removal in a case of autosomal recessive bestrophinopathy.	Moreira, C.A., Jr.; Moreira-Neto, C.A.; Junqueira Nobrega, M.; Cunha de Souza, E.	Case Rep Ophthalmol **2017**, 8, 265-270.
Best1 protein stability and degradation pathways differ between autosomal dominant best disease and autosomal recessive bestrophinopathy accounting for the distinct retinal phenotypes.	Milenkovic, A.; Milenkovic, V.M.; Wetzel, C.H.; Weber, B.H.F.	Hum Mol Genet **2018**, 27, 1630-1641.

Normal electrooculography in best disease and autosomal recessive bestrophinopathy.	Khan, K.N.; Islam, F.; Holder, G.E.; Robson, A.; Webster, A.R.; Moore, A.T.; Michaelides, M.	Retina **2018**, 38, 379-386.

The articles' list is not exhaustive.

Table S2. List of all *BEST1* variants detected in affected individuals of each family.

	Exon 1	Exon2	Exon 3	Exon 4	Exon 5	Exon 6	Exon 7	Exon 8	Exon 9	Exon 10	Exon 11
**F1: III.2**	No	No	homozygous c.209A>G p.Asp70Gly rs749295558; disease causing; moderately conserved; MAF: 0.00002 (ExAc) 0.00008 (GnomAD) (never homozygous)	No	No	No	No	No	No	homozygous c.1230G>A, p.Thr410=, rs149698; SNP; not conserved; Highest population MAF: 0.38	homozygous.1740-42T>G, intronic, rs195155; SNP; not conserved; Highest population MAF: 0.04
**F1: IV.1**	No	heterozygous c.109T>C p.Leu37=, rs1800007; SNP; not conserved; Highest population MAF: 0.49	heterozygous c.209A>G p.Asp70Gly rs749295558; disease causing; moderately conserved; MAF: 0.00002 (ExAc) 0.00008 (GnomAD) (never homozygous)	No	No	No	No	No	No	heterozygous, c.1403C>T , p.Pro468Leu, rs747043918, disease causing; very conserved; MAF: 0.000008 (ExAc) 0.000004 (GnomAD) (never homozygous)<br><br>heterozygous c.1230G>A, p.Thr410=,	heterozygous. 3'UTR, g. 61964338 T>C, rs1801327, SNP; not conserved

										rs149698; SNP; not conserved; Highest population MAF: 0.38	
F1: IV.3	N.S	N.S	heterozygous c.209A>G p.Asp70Gly rs749295558; disease causing; moderately conserved; MAF: 0.00002 (ExAc) 0.00008 (GnomAD) (never homozygous)	N.S	N.S	N.S	N.S	N.S	N.S	heterozygous, c.1403C>T , p.Pro468Leu, rs747043918, disease causing; very conserved; MAF: 0.000008 (ExAc) 0.000004 (GnomAD) (never homozygous)	N.S
**F2: IV.1**	No	homozygous c.109T>C p.Leu37=, rs1800007; SNP; not conserved; Highest population MAF: 0.49	No	No	No	homozygous c.696C>A p.Ile232=, rs1805140; SNP; not conserved; Highest population MAF: 0.25	homozygous c.830C>T p.Thr277Met rs775791299; disease causing; very conserved; MAF: 0.0000082 (ExAc) 0.0000082 (GnomAD) (never homozygous)	No	No	No	homozygous c.1608T>C p.Thr536=, rs1800009, SNP; not conserved; Highest population MAF: 0.49.<br><br>rs195155 (same as F1: III.2).
**F34:**	homozygous,	homozygous	No	No	No	No	No	No	No	homozygous,	No

**II.1**	5'UTR, g.61950243; (c.-221) T>C, rs972353, SNP; not conserved, Highest population MAF: 0.50	c.109T>C p.Leu37=, rs1800007; SNP; not conserved; Highest population MAF: 0.49						c.1403C>T , p.Pro468Leu, rs747043918, disease causing; very conserved; MAF:0.000008 (ExAc) 0.000004 (GnomAD) (never homozygous)	

N.S: not screened.

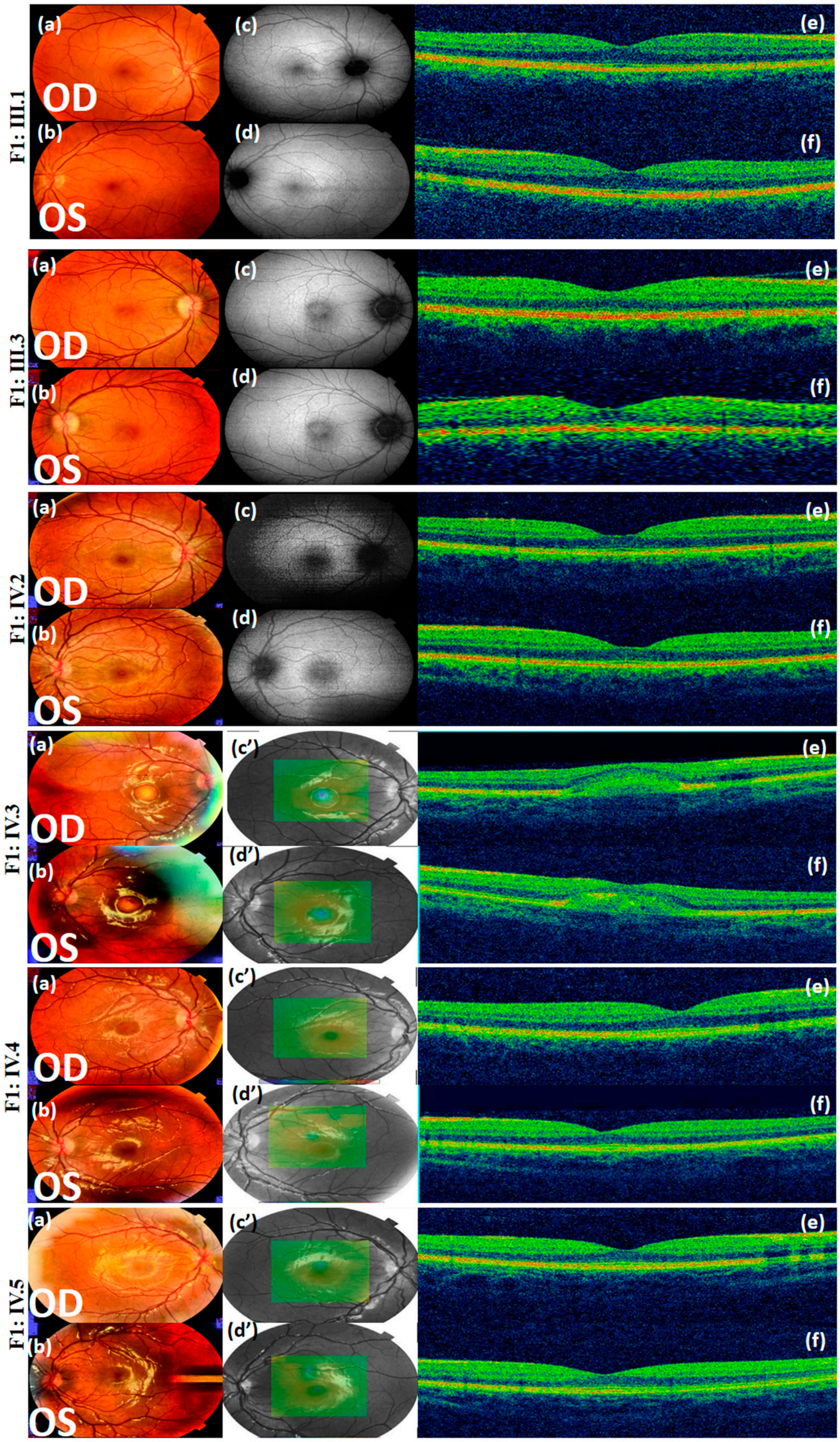

Figure S1. Color fundus photographs (a and b), auto-fluorescence pictures (c and d), red free fundus photographs (c′ and d′), optical coherence tomography scans; OCT (e and f) of family members (F1: III.1, F1: III.3, F1: IV.2, F1: IV.3, F1: IV.4, F1: IV.5). OD= oculus dexter; OS= oculus sinister.

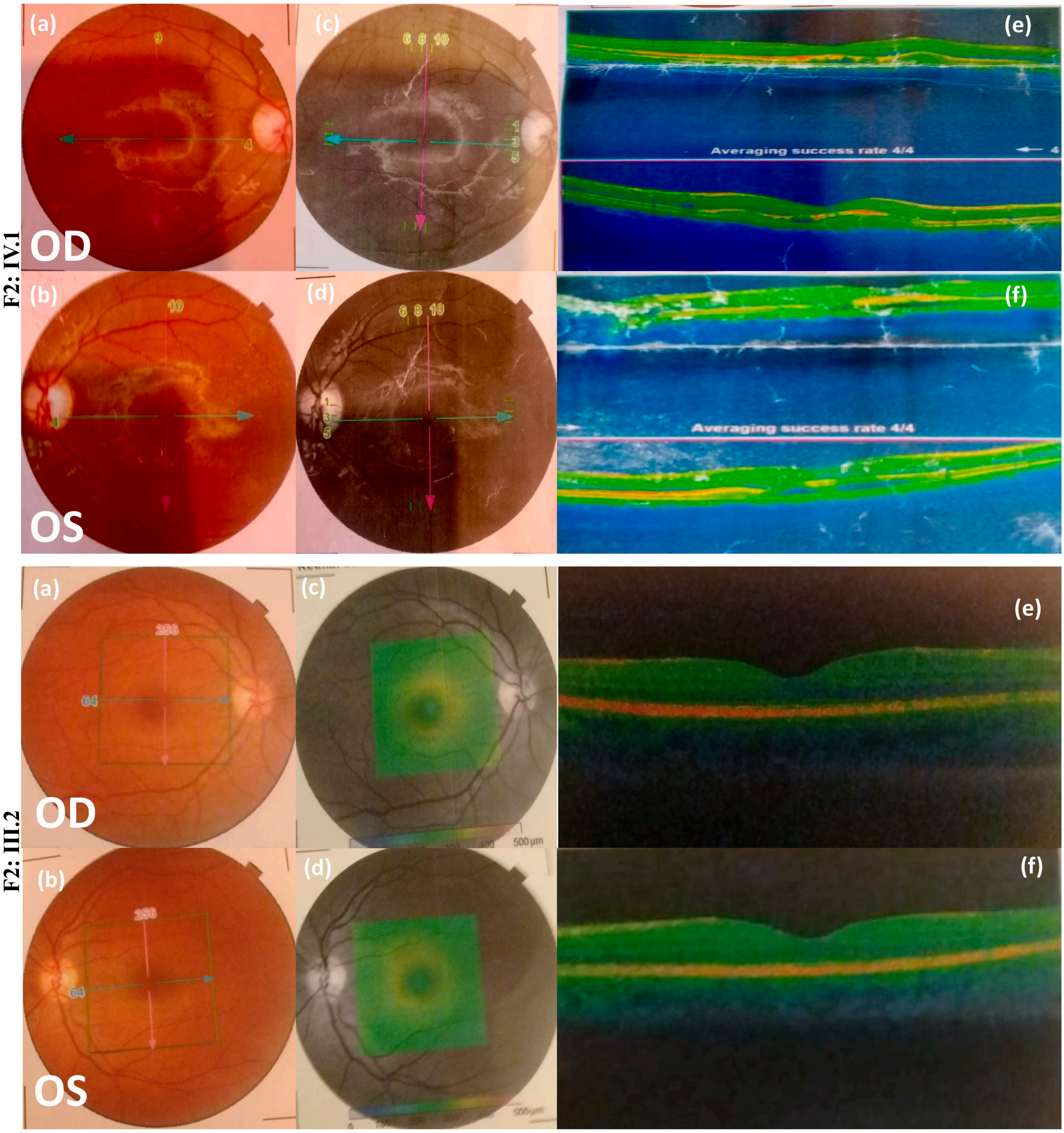

Figure S2. Color fundus photographs (a, b, c, d), optical coherence tomography scans; OCT; (e, f) of index F2: IV.1. OD= oculus dexter; OS= oculus sinister.

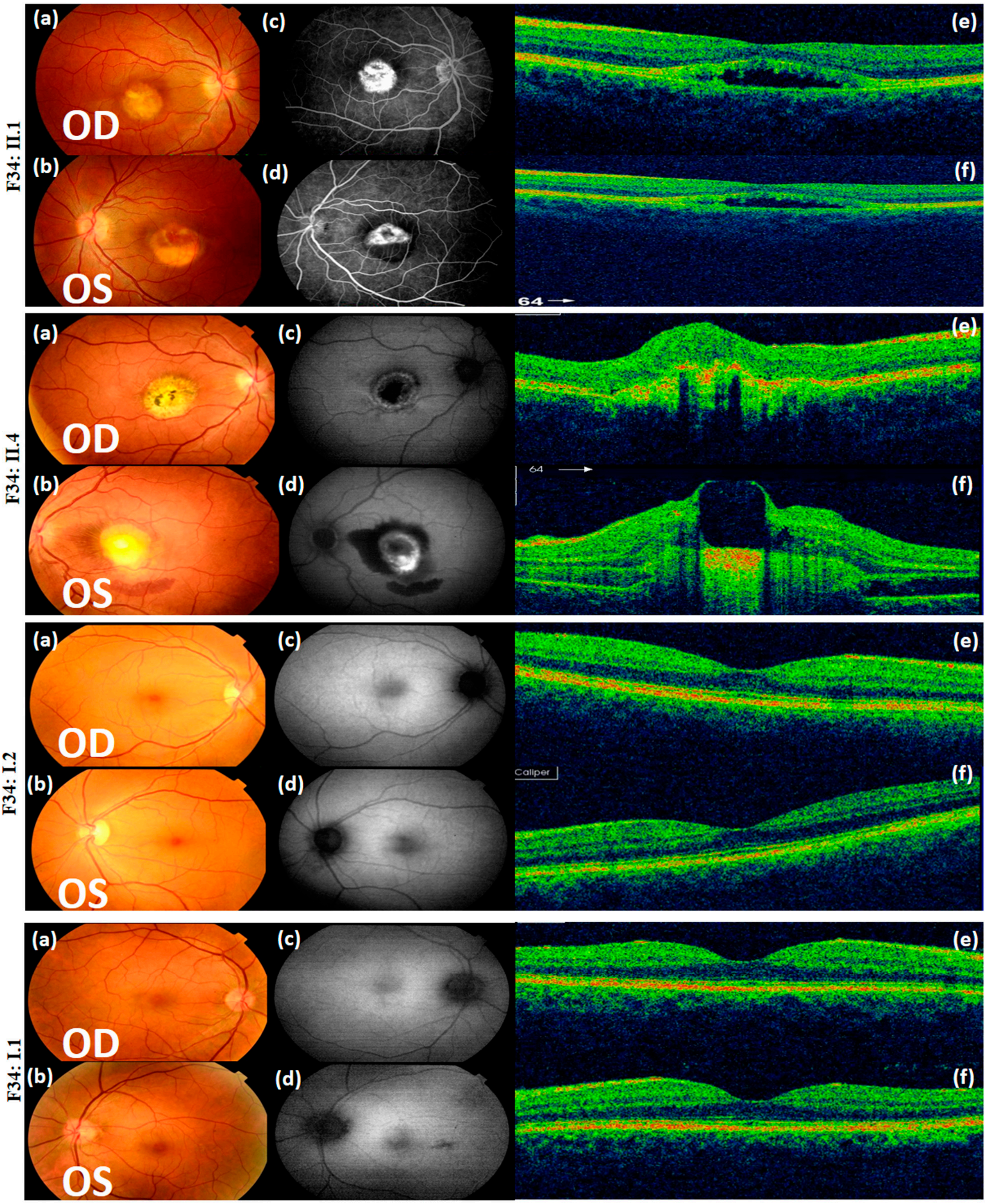

Figure S3. Color fundus photographs (a and b), auto-fluorescence pictures; (c and d), optical coherence tomography scans; OCT; (e and f) of indexes (F34: II.1 - F34: II.4) and their parents (F34: I.2 - F34: I.1). OD = oculus dexter; OS= oculus sinister.

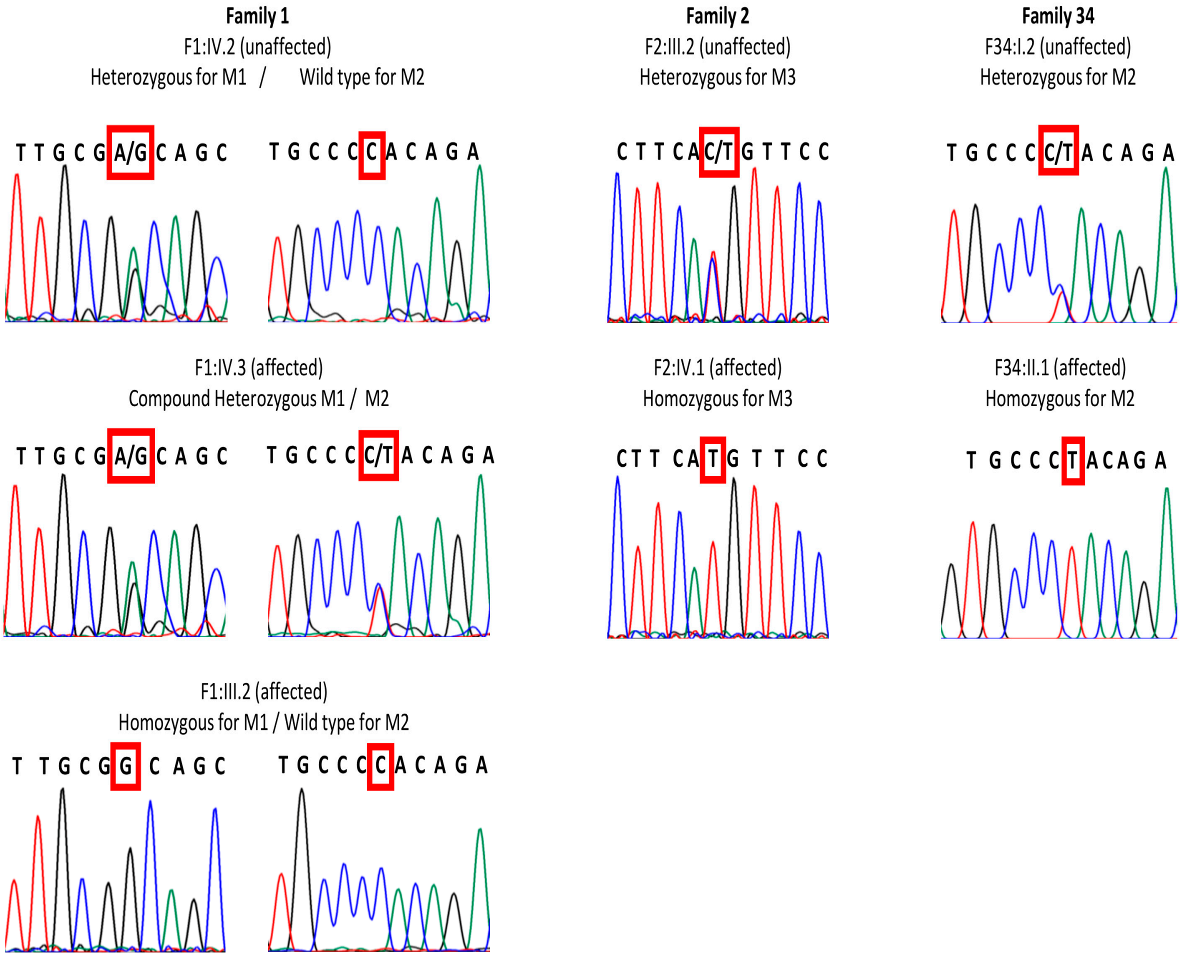

Figure S4. Chromatograms of additional affected and unaffected family members from Families 1, 2 and 34. .
